# Recommendations from the COST action CA17116 (SPRINT) for the standardization of perinatal derivative preparation and *in vitro* testing

**DOI:** 10.3389/fbioe.2023.1258753

**Published:** 2023-11-14

**Authors:** Aleksandar Janev, Asmita Banerjee, Adelheid Weidinger, Jure Dimec, Brane Leskošek, Antonietta Rosa Silini, Tina Cirman, Susanne Wolbank, Taja Železnik Ramuta, Urška Dragin Jerman, Assunta Pandolfi, Roberta Di Pietro, Michela Pozzobon, Bernd Giebel, Günther Eissner, Polonca Ferk, Ingrid Lang-Olip, Francesco Alviano, Olga Soritau, Ornella Parolini, Mateja Erdani Kreft

**Affiliations:** ^1^ Institute of Cell Biology, Faculty of Medicine, University of Ljubljana, Ljubljana, Slovenia; ^2^ Ludwig Boltzmann Institute for Traumatology, The Research Center in Cooperation with AUVA, Vienna, Austria; ^3^ Austrian Cluster for Tissue Regeneration, Vienna, Austria; ^4^ ELIXIR-SI Centre, Faculty of Medicine, Institute for Biostatistics and Medical Informatics, University of Ljubljana, Ljubljana, Slovenia; ^5^ Centro di Ricerca E. Menni, Fondazione Poliambulanza, Brescia, Italy; ^6^ Cryobiology Centre, Department of Therapeutic Services, Blood Transfusion Centre of Slovenia, Ljubljana, Slovenia; ^7^ Department of Medical, Oral and Biotechnological Sciences, Center for Advanced Studies and Technology—CAST, “G. d’Annunzio” University of Chieti-Pescara, Chieti, Italy; ^8^ Department of Medicine and Aging Sciences, “G. d’Annunzio” University of Chieti-Pescara, Chieti, Italy; ^9^ Department of Women’s and Children’s Health, University of Padova, Padova, Italy; ^10^ Foundation Institute of Pediatric Research Città della Speranza, Padova, Italy; ^11^ Institute for Transfusion Medicine, University Hospital Essen, University Duisburg-Essen, Essen, Germany; ^12^ Systems Biology Ireland, School of Medicine, Conway Institute, University College Dublin, Dublin, Ireland; ^13^ Division of Cell Biology, Histology and Embryology, Gottfried Schatz Research Center, Medical University of Graz, Graz, Austria; ^14^ Department of Biomedical and Neuromotor Sciences, University of Bologna, Bologna, Italy; ^15^ Laboratory of Tumor Cell Biology and Radiobiology, Institute of Oncology “Prof. Dr. Ion Chiricuta”, Cluj-Napoca, Romania; ^16^ Department of Life Science and Public Health, Università Cattolica del Sacro Cuore, Rome, Italy; ^17^ Fondazione Policlinico Universitario “Agostino Gemelli” IRCCS, Rome, Italy

**Keywords:** standardization, perinatal derivatives, recommendations, CA17116 (SPRINT), PnD e-questionnaire

## Abstract

Many preclinical studies have shown that birth-associated tissues, cells and their secreted factors, otherwise known as perinatal derivatives (PnD), possess various biological properties that make them suitable therapeutic candidates for the treatment of numerous pathological conditions. Nevertheless, in the field of PnD research, there is a lack of critical evaluation of the PnD standardization process: from preparation to *in vitro* testing, an issue that may ultimately delay clinical translation. In this paper, we present the PnD e-questionnaire developed to assess the current state of the art of methods used in the published literature for the procurement, isolation, culturing preservation and characterization of PnD *in vitro*. Furthermore, we also propose a consensus for the scientific community on the minimal criteria that should be reported to facilitate standardization, reproducibility and transparency of data in PnD research. Lastly, based on the data from the PnD e-questionnaire, we recommend to provide adequate information on the characterization of the PnD. The PnD e-questionnaire is now freely available to the scientific community in order to guide researchers on the minimal criteria that should be clearly reported in their manuscripts. This review is a collaborative effort from the COST SPRINT action (CA17116), which aims to guide future research to facilitate the translation of basic research findings on PnD into clinical practice.

## 1 Introduction

Over the past two decades, there has been an increased understanding of the therapeutic mechanisms of action (MoA) of perinatal derivatives (PnD), defined as birth-associated tissues, isolated cells and the factors secreted by these cells (in their entirety referred to as secretome), such as free-floating factors, extracellular vesicles (EVs), and extracellular matrix components (Silini et al., 2020). Indeed, many preclinical studies have now demonstrated that PnD can restore tissue damage and promote regeneration and repair of the tissue microenvironment. Despite the fact that a variety of PnD have been investigated in preclinical regenerative medicine research, there is still the lack of knowledge of the precise mechanisms of how PnD work therapeutically, which is why their translation into clinical practice is slow.

Many issues have contributed to the sluggish clinical translation of PnD such as aspects related to preclinical testing (e.g., animal models, dose, and administration route) and patients (e.g., comorbidities), yet upstream parameters such as disparities and inconsistencies in PnD definition are critical and perhaps underestimated. These critical factors include a precise description of the PnD used and adequate information on the *in vitro* tests used to characterize PnD. A recent study by Sabol et al. (2022), reported that published data from placental tissues in their final, useable form is lacking. In their study they highlighted the need for standardized data collection and reporting in the field of dry membrane allografts. They presented standardized characterization of hAM and chorion and argue that this is critical for methods applicable to both *in vitro* and clinical studies (Sabol et al., 2022). Norte-Muñoz et al. (2022), aimed to investigate the current level of standardization of applied technical procedures in preclinical studies using various PnDs. They argued that due to lack of standardization between the studies, which were selected following a systematic PubMed search, there is no clear consensus regarding the status of the administered PnD and their mode of action (Norte-Muñoz et al., 2022). In addition, Papait et al. (2022) underline the lack of PnD potency assays and the need for their standardization. Thus, the identification of critical points of *in vitro* protocols for the procurement, isolation, culture, preservation, and characterization of PnD is fundamental for standardization and for comparison of results obtained from different laboratories.

Among others, detailed information on the health status of the donor is required. For example, PnD obtained from women with diabetes mellitus, gestational diabetes mellitus or from women who are obese or have a higher risk of preterm/premature rupture of membranes, a pregnancy complication associated with weakening of the fetal membranes may have altered properties when compared to PnD obtained from healthy mothers. In addition, specific health issues, such as those mentioned, can alter the inflammatory status both of the mother and the newborn (Castellana et al., 2018; Corrêa-Silva et al., 2018; Ucci et al., 2019), thus underlining the importance in claiming the health status of the mother and, more in general, in identifying also other critical aspects that must be addressed when describing protocols used to isolate and characterize the PnD. Another example is the type of stimuli used when investigating the *in vitro* immunomodulatory properties of PnD that can act via different pathways and can ultimately impact the degree to which PnD are able to modulate immune cells (Papait et al., 2022).

Thus, to better address clinical translation, reproducibility, and transparency in the field of PnD research, the expert scientific community must establish minimal criteria to report first and foremost the PnD origin, isolation, preparation, culture and *in vitro* characterization. The absence of a consensus will lead to ongoing difficulties in the comparison of different studies, extrapolation from study findings, and can also affect results of preclinical studies, ultimately resulting in controversy outcomes among clinical trials and selected preclinical studies.

Our SPRINT COST Action has recently published a series of consensus reviews on the relevance and potency of classical and new *in vitro* assays to demonstrate efficacy and potential MoA of PnD (Flores et al., 2022; Papait et al., 2022; Pozzobon et al., 2022; Silini et al., 2022). Given the strong representation of PnD experts in the SPRINT COST Action, we now seek to address the issue of PnD definition and their characterization and provide a checklist of criteria that should be considered for any future study regarding PnD isolation, characterization and *in vitro* testing.

## 2 A tool for PnD standardization—the development of the PnD e-questionnaire

In order to evaluate the current state of conditions (state-of-the-art methods used for *in vitro* characterization), the PnD e-questionnaire was developed and set up. It is intended to serve as a guide for the scientific community, listing the relevant information that should be provided when researching PnD, thus facilitating the exchange of information and comparison of data.

At first, we developed a checklist based on information that our SPRINT COST Action considered relevant regarding the reporting on PnD, such as the definition of their tissue of origin, collection, preparation, isolation, preservation, and characterization. The e-questionnaire was used to screen PnD publications to gather information on the current state of conditions.

The PnD e-questionnaire was developed as a web-based electronic data capture form by using the Research Electronic Data Capture (REDCap) application which was used also for data management. REDCap is a web-based application developed by Vanderbilt University to capture data for clinical research and create databases and projects (Harris et al., 2009; Harris et al., 2019). It is Health Insurance Portability and Accountability Act (HIPAA) compliant, secure and intuitive to use. The development of interactive data entry e-questionnaires is workflow-based and enables multiple branches based on selected conditions or answers. Collected data can be exported to statistical programs and other data analysis software. REDCap is designed to provide a secure environment so that research teams can collect and store highly sensitive information. The main creative value of developed PnD e-questionnaire is the generated workflow/process and data model that could be used with any other advanced electronic data capture software. Web based solution enables easy multisite, simultaneous and geographically dispersed data collection.

For the questions we used many different functions and field types in the data model such as multiple choice (radio or checkbox), yes/no and text field types. The data were collected from the members of the SPRINT COST Action between March 2019 and October 2022. Regular invitations and reminders were sent out to increase the number of collected questionnaires. The data used in the report were from members who submitted data and agreed that the information provided could be made publicly available.

### 2.1 The PnD e-questionnaire structure

**FIGURE 1:**
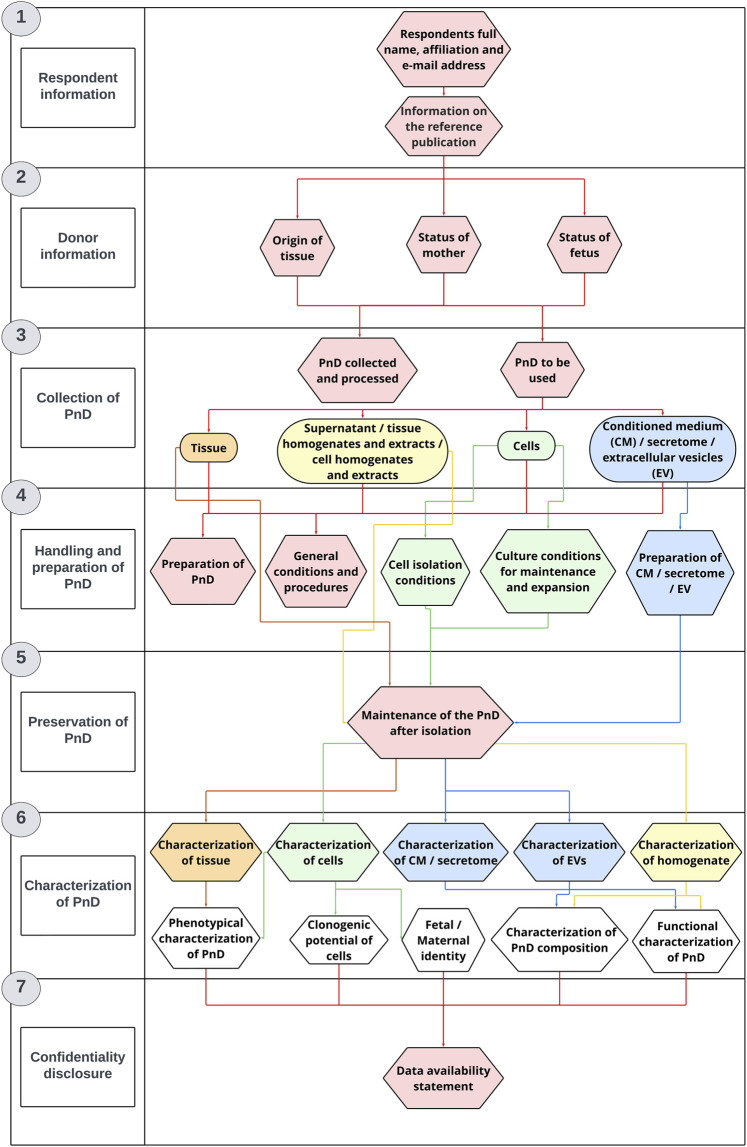
The questionnaire structure. The PnD e-questionnaire was applied for PnD characterization and *in vitro* research.

The PnD e-questionnaire is divided into seven main sections: Information about the respondent, Information about the donor, Collection of PnD, Handling and preparation of PnD, Preservation of PnD, Characterization of PnD and Confidentiality disclosure (Figure 1).

#### 2.1.1 Respondent information

The first section of the PnD e-questionnaire contains questions that refer to the personal information of the respondents that includes name, affiliation and email address. In case the research project using PnD was already published, the respondents were invited to provide additional details regarding the publication.

#### 2.1.2 Donor information

The first part of the second section of the PnD e-questionnaire contains questions regarding the ethical approval and the informed consent. The second part of this section contains general questions about the status of the donor. Participants are asked to provide information on the donor’s ethnicity, age group, previous medical conditions and pharmacological treatment during pregnancy. They are also asked to indicate whether the donor had been tested for HIV, hepatitis B and C, syphilis, toxoplasma, West Nile virus, Zika virus and SARS-CoV-2 virus. This section also includes questions about the method of delivery (vaginal or caesarean section). The last part of the second section includes general questions about the status of the newborn. Namely, the participants are kindly asked to provide information on the gender and gestational age at the time of the PnD isolation. Legislative requirements regarding donor selection, tissue procurement and mandatory testing of donated PnD are described in more detail in the Supplementary Material.

#### 2.1.3 Collection of PnD

In the third section of the PnD e-questionnaire, participants are asked to provide information on the type of perinatal tissue that was collected and processed. They are also asked to specify which PnD preparations were used for *in vitro* research, such as supernatant, tissue homogenates and extracts, cell homogenates and extracts, tissue, conditioned medium (CM), secretome or EV.

#### 2.1.4 Handling and preparation of PnD

The fourth section includes questions referring to the handling and preparation of the selected PnD after isolation. In the first part, participants are asked to provide additional information on the general conditions and procedures used, e.g., the method of tissue decontamination, homogenization and extraction. The second part of the fourth section contains questions on cell isolation protocols. Participants are also asked to provide information on the general conditions for maintaining and expanding cells.

#### 2.1.5 Preservation of PnD

The fifth section of the PnD e-questionnaire contains questions on the maintenance of the PnD after the processing step. Participants are asked to provide information on processing protocols, including detailed description of cryopreservation, lyophilization, dehydration, freeze-drying or cell culturing protocols.

#### 2.1.6 Characterization of PnD

The first part of the sixth section of the PnD e-questionnaire includes questions related to the characterization of the previously selected PnD. Participants are asked to provide detailed information on which methods were performed to analyze the expression of genes and small RNAs, protein content, lipid and metabolic profile. The second part of the sixth section contains questions on the functional characterization of the selected PnD. Participants are asked to provide detailed information on the functional assays used to monitor various cellular processes such as mitochondrial activity, cell proliferation, cell death, cell differentiation, angiogenesis, migration, invasion and microbial growth. Finally, participants are asked to indicate which positive and negative controls were included in their functional assays.

#### 2.1.7 Confidentiality disclosure

In the last section of the PnD e-questionnaire, participants are asked to confirm whether the information they provided was correct. The information provided is used only for statistical analysis which is made available to the public (name and surname of participants are anonymized and not included in the statistical analysis or used for any other purpose).

## 3 Results

The enquiry to complete the PnD e-questionnaire was sent to 93 members of SPRINT COST Action (working groups 1–5), 35.5% of which completed the e-questionnaire. The data analyzed in this study originates from 60 biological donors, and it has been verified by the respondents that this data is sourced exclusively from published peer-reviewed articles.

### 3.1 Legal requirements for the use of human tissue in research

**FIGURE 2:**
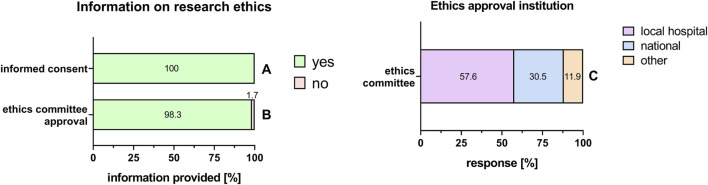
Information on research ethics. Percentage of respondents providing information (yes = green/no = pink) on informed consent **(A)**, ethics committee approval **(B)**, and information on ethics approval institution **(C)**. The percentages of respondents are indicated within or right above each bar.

In the PnD e-questionnaire, we first asked whether information about the fulfillment of legal requirements was included in the Materials and Methods section of publications. Working with substances of human origin, it is mandatory to have a signed informed consent which clearly states that the tissue donation is intended for research. All the respondents stated that an informed consent was obtained from the mothers to donate their placentas for research (Figure 2A). In addition, 98.3% were able to provide information on the ethics committee approval for research (Figure 2B), from which the majority was obtained from local hospital ethics committees (57.6%), and the minor part from a national ethics committee (30.5%) (Figure 2C).

PnD are highly variable biological materials. Therefore, it is important to use as many measures as possible to lower the influence of material inherent factors on the experiment or final PnD product, respectively. To ensure comparability of research data, information on a number of factors that might influence the regenerative potential of the PnD is required.

### 3.2 Information about the donor

**FIGURE 3:**
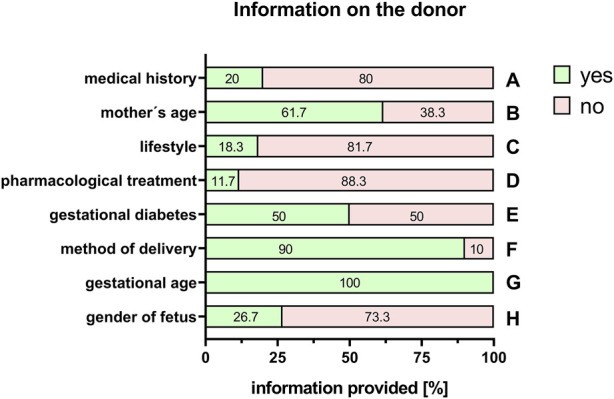
Information on the donor. Percentage of respondents providing information (yes = green/no = pink) on the medical history of the mother **(A)**, the mother’s age **(B)**, lifestyle **(C)**, pharmacological treatments **(D)**, gestational diabetes **(E)**, method of delivery **(F)**, gestational age of the fetus **(G)**, and the gender of the fetus **(H)**. The percentages of respondents are indicated within each bar.

With the exception of the mother’s age (61.7%, Figure 3B), information on the mother’s pre-existing medical history was scarce (20%; Figure 3A). Information on significant life-style factors, such as smoking, drug/alcohol abuse or obesity was rarely provided (18.3%; Figure 3C). This was also the case with known pharmacological treatments during pregnancy (11.7%, Figure 3D). Information on pregnancy-related diseases, for example, gestational diabetes, was also often not included in the selection of donors in 50% (Figure 3E). The method of delivery (vaginal or caesarean section) was stated in 90% of the cases (Figure 3F). Regarding information on the fetus, respondents provided full information on the gestational age (100%, Figure 3G). However, only 26.7% provided information about the gender of the newborn (Figure 3H).

**FIGURE 4:**
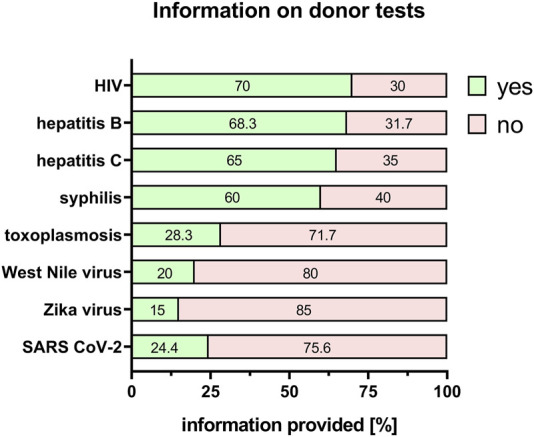
Information on donor tests. Percentage of respondents providing information (yes = green/no = pink) on HIV, hepatitis B/C, syphilis, toxoplasmosis, West Nile virus, Zika virus and SARS CoV-2 status. The percentages of respondents are indicated within each bar. HIV = human immunodeficiency virus; SARS CoV-2 = severe acute respiratory syndrome coronavirus 2.

Besides influencing the properties and potency of the tissue, any bacterial or viral infection of the donor poses a potential risk to those working with the donor material, as well as to midwives and doctors involved. Furthermore, there is a possibility that if the mother is infected, the newborn baby could also be at risk. Therefore, the donor should ideally be tested for human immunodeficiency virus (HIV), hepatitis B and C, syphilis, toxoplasmosis, West Nile virus, Zika virus and severe acute respiratory syndrome coronavirus type 2 (SARS-CoV-2) virus. Information about a negative or positive result of the mother/donor test was provided for HIV (70%), hepatitis B (68.3%), hepatitis C (65%), syphilis (60%), toxoplasmosis (28.3%), West Nile virus (20%), Zika virus (15%) and SARS-CoV-2 virus (24.4%) (Figure 4). The others could not provide any test results as either the donor was not tested or the researchers did not have information about the test results (Figure 4).

### 3.3 Information on collection/procurement of the tissue

**FIGURE 5:**
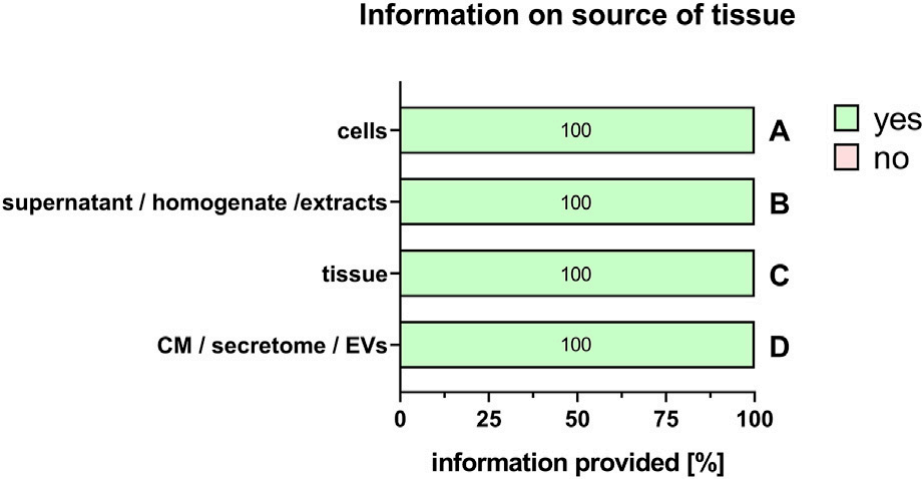
Information on the source of the tissue. Percentage of respondents providing information (yes = green/no = pink) on the source of the tissue when using cells **(A)**, supernatant/homogenate/extracts **(B)**, tissue **(C),** and conditioned medium/secretome/EVs **(D)**. The percentages of respondents are indicated within each bar. CM, conditioned medium; EVs, extracellular vesicles.

**FIGURE 6:**
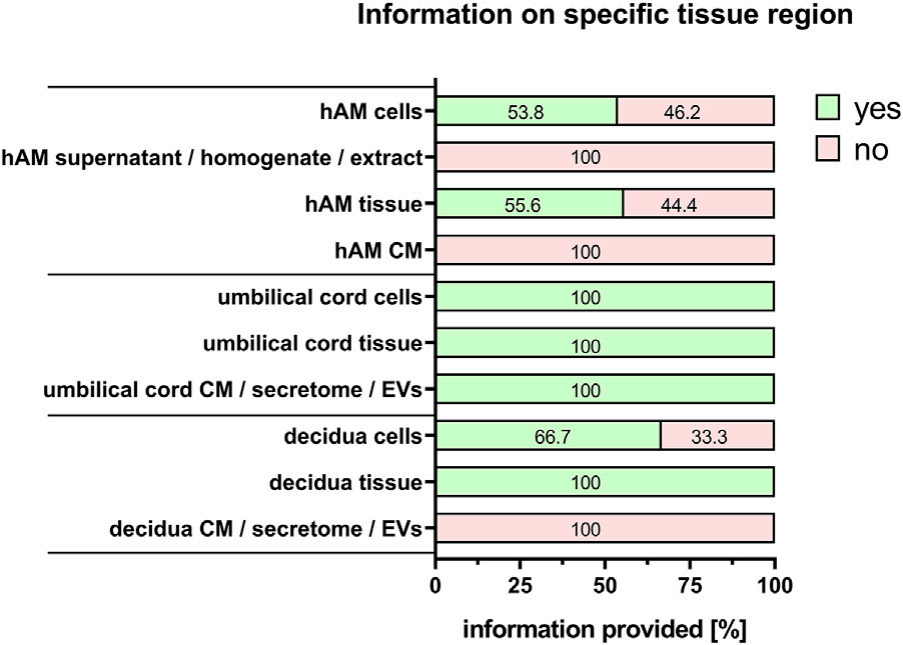
Information on specific tissue regions. Percentage of respondents providing information (yes = green/no = pink) on the specific tissue region when isolating/preparing cells, supernatant, homogenate, extract, tissue, CM, secretome and EV. The percentages of respondents are indicated within each bar. CM, conditioned medium; EVs, extracellular vesicles.

For the comparability of results, it is also important to provide information on the type of perinatal tissue used for PnD preparation. We recently published a consensus paper on the identification of different perinatal tissues and the cells isolated from these tissues, along with their nomenclature (Silini et al., 2020). Indeed, there are many different perinatal tissues, which include human placenta, human chorion, human amniotic membrane, human umbilical cord, human amniotic fluid and human decidua. For all PnD used, the specific tissue type was defined in 100% of the cases (Figure 5). However, information on the specific tissue region from which the cells, supernatant, homogenate, extract, tissue, CM, secretome or EVs of interest were isolated, was fully provided only for human umbilical cord PnD (Figure 6).

### 3.4 Information on isolation/preparation of PnD

**FIGURE 7:**
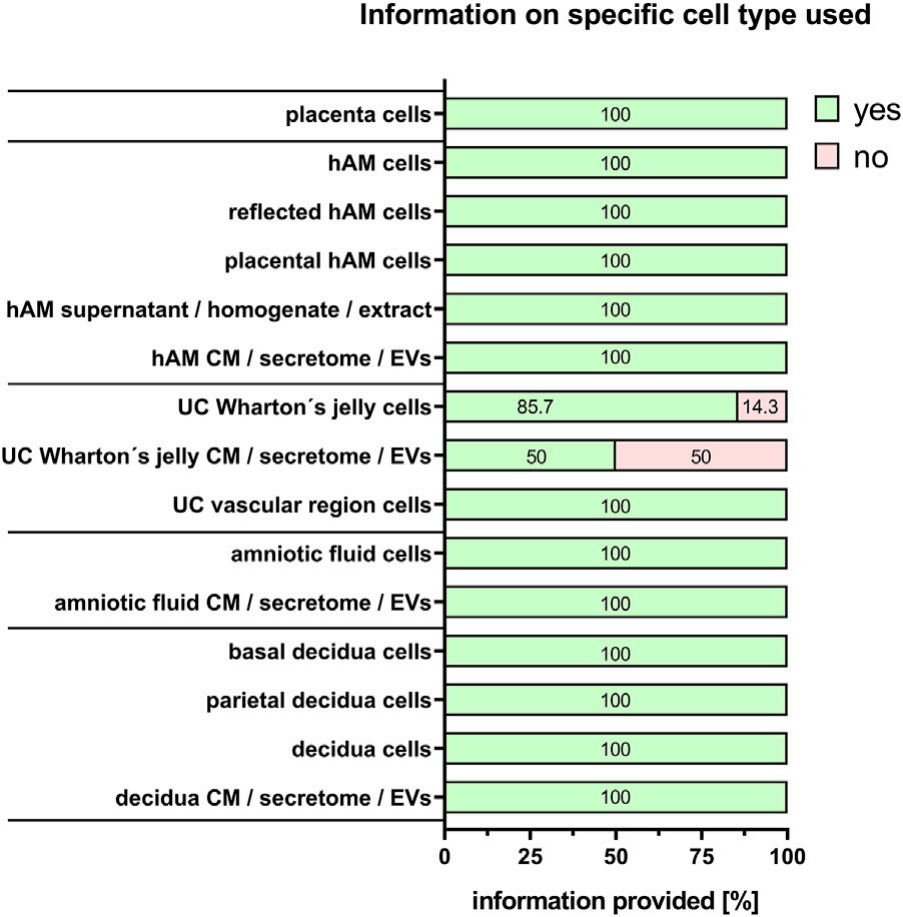
Information on specific cell type used. Percentage of respondents providing information (yes = green/no = pink) on the specific cell type used. The percentages of respondents are indicated within each bar. hAM, human amniotic membrane; CM, conditioned medium; EVs, extracellular vesicles; UC, umbilical cord.

**FIGURE 8:**
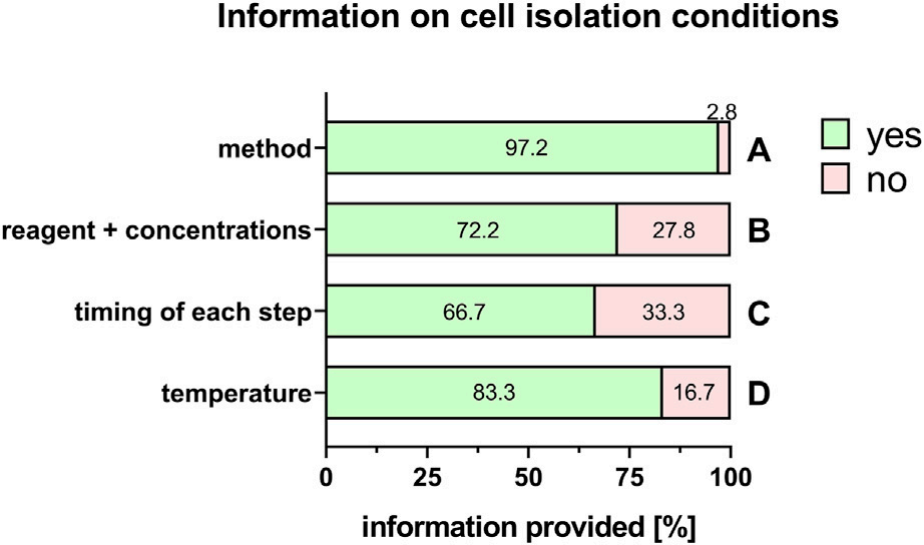
Information on cell isolation conditions. Percentage of respondents providing information (yes = green/no = pink) on isolation method **(A)**, reagents + concentrations **(B)**, timing of each step **(C)** and temperature **(D)**. The percentages of respondents are indicated within or right above each bar.

Commonly used PnD in research are tissues, isolated cells, supernatant/tissue and extracts/cell homogenate preparations, and conditioned medium/secretome/EVs preparations. “Cells” was the largest group stated by the respondents (60%), and is explored in more detail. For isolated cells from different tissues, almost all respondents provided detailed information on the specific cell types used (100%) except when human umbilical cord Wharton´s jelly-derived cells were used either directly (86.7%), or for the generation of conditioned medium/secretome/EVs (50%) (Figure 7). Regarding cell isolation protocols, specific information was provided on the method for cell isolation (97.2%, Figure 8A), specific reagents and their concentrations (72.2%, Figure 8B), timing of each isolation step (66.7%, Figure 8C) and the specific temperature (86.1%, Figure 8D).

**FIGURE 9:**
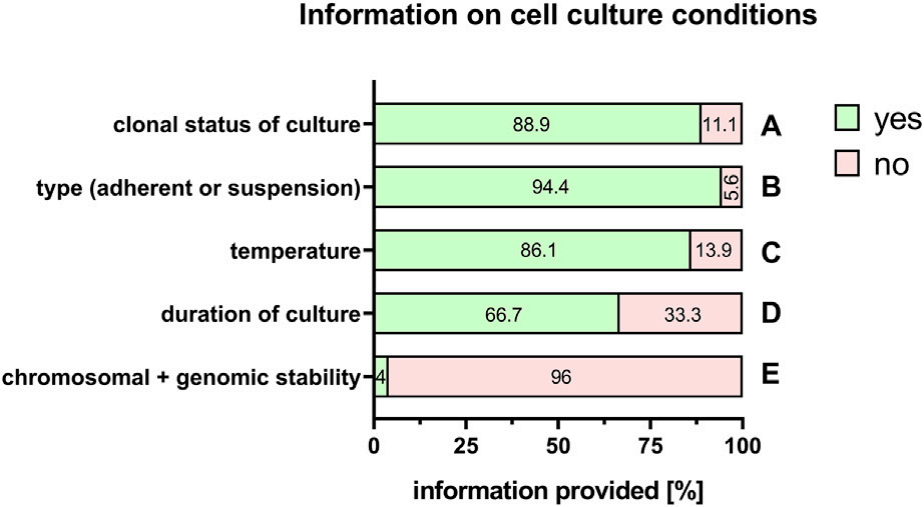
Information on cell culture conditions. Percentage of respondents providing information (yes = green/no = pink) on clonal status of the culture **(A)**, adherent or suspension culture **(B)**, temperature **(C)**, duration of the culture **(D)**, and chromosomal and genomic stability **(E)**. The percentages of respondents are indicated within each bar.

Even more important are the culture conditions for the maintenance and expansion of cells. Here, among others, information was available on clonal status of the cell culture (88.9%, Figure 9A), type of cell culture (adherent or in suspension) (94.4%, Figure 9B), specific temperature for cell incubation (86.1%, Figure 9C), duration of cell culture (66.7%, Figure 9D), and chromosomal and genomic stability (4%, Figure 9E).

### 3.5 Information on characterization

**FIGURE 10:**
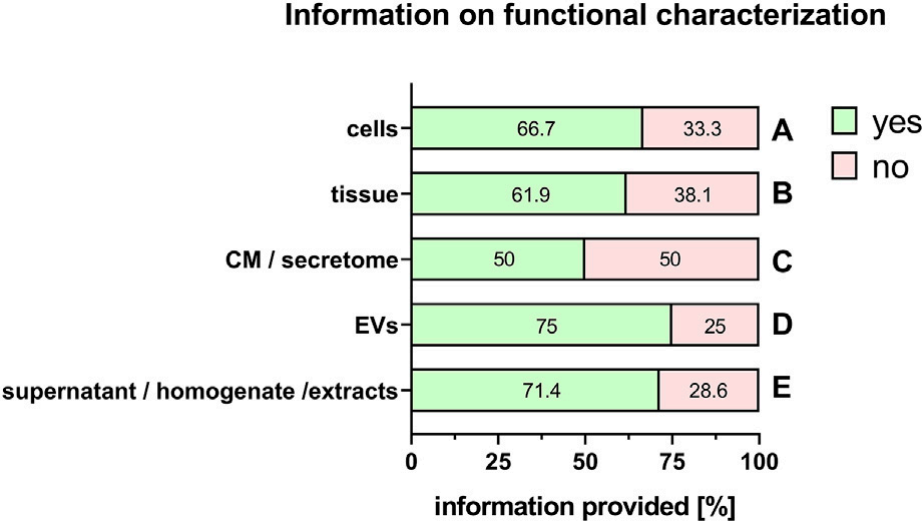
Information on functional characterization of PnD. Percentage of respondents providing information (yes = green/no = pink) on functional characterization of cells **(A)**, tissue **(B)**, conditioned medium (CM)/secretome **(C)**, extracellular vesicles (EV) **(D)**, and supernatant/homogenate/extracts **(E)**. The percentages of respondents are indicated within each bar.

It is also important to provide adequate information on the characterization of the PnD, for example, phenotypical characterization of tissue or fetal/maternal identity and clonogenic potential of cells (Table 1). Furthermore, the molecular composition of conditioned medium, secretome, supernatant, homogenate, extracts and EVs should be characterized (Table 1). In addition, when stating information on the functional characteristics of the PnD used, it is important to define the *in vitro* mode of action that was tested for (Table 1). Information of functional characteristics was provided for cells (66.7%, Figure 10A), tissue (61.9%, Figure 10B), conditioned medium/secretome (50%, Figure 10C), extracellular vesicles (75%, Figure 10D), and supernatant/homogenate/extracts (71.4%, Figure 10E).

**TABLE 1:** Recommendations for the characterization of perinatal derivatives. Suggested characteristics to be analyzed according to the PnD e-questionnaire.

Characterization of PnD		Tissue	Cells	CM secretome	EVs	Supernatant homogenate extract
Phenotypic characterization						
Morphology		✓	✓			
Histology		✓				
Gene expression profile		✓	✓			
Protein expression profile		✓	✓			
Lipid profile		✓	✓			
Metabolic profile		✓	✓			
Epigenetic profile		✓	✓			
miRNA analysis		✓	✓			
siRNA analysis		✓	✓			
Functional characterization						
Mitochondrial activity		✓	✓	* ✓	* ✓	* ✓
Cell proliferation		✓	✓	* ✓	* ✓	* ✓
Cell death		✓	✓	* ✓	* ✓	* ✓
Cell migration/invasion		✓	✓	* ✓	* ✓	* ✓
Microbial growth		✓	✓	* ✓	* ✓	* ✓
Angiogenesis		✓	✓	* ✓	* ✓	* ✓
Differentiation status		✓	✓	* ✓	* ✓	
Immunological characterization		✓	✓	* ✓	* ✓	* ✓
Immunogenicity		✓	✓	* ✓	* ✓	* ✓
Immunomodulation		✓	✓	* ✓	* ✓	* ✓
Cytokine production		✓	✓	* ✓	* ✓	* ✓
Fetal/maternal identity						
Genetic identity			✓			
Clonogenic potential						
Marker analysis			✓			
Proliferation marker			✓			
Characterization of composition						
Gene expression profile					✓	✓
Protein expression profile				✓	✓	
Protein content profile						✓
Lipid profile				✓	✓	✓
Metabolic profile				✓	✓	✓
miRNA analysis				✓	✓	✓
siRNA analysis				✓	✓	✓
Size ranges					✓	
Density					✓	
Refractive index					✓	

*Characterization of target cells/tissue; PnD, perinatal derivatives; CM, conditioned medium; EVs, extracellular vesicles.

## 4 Current obstacles and future recommendations for PnD *in vitro* standardization

Although huge efforts are put into PnD research all over the world, merely a fraction of this knowledge base, from which both medical staff and patients could benefit greatly, is translated into clinics. The reasons for this are manifold and primarily due to an incomplete description of the PnD used or the lacking of detailed information on the PnD process of characterization. Collaboration between medical doctors and researchers is fundamental when performing research with biological samples. On one hand, the availability of clinical data significantly helps us pinpoint (and possibly minimize) biological variability and understand better our data, on the other hand, a clear, detailed protocol facilitates the comparison of data between different groups.

This poses a challenge for researchers working with donor materials. High donor variability and limited access to donor material are typical extrinsic factors that can already exacerbate any study. In addition, if certain detrimental intrinsic factors come into play, the reproducibility and standardization of results, and, consequently, the quality of a clinical product, can be hampered to a great extent. Obviously, the development of a medicinal product undergoes many levels, starting from *in vitro* testing to animal studies to the preclinical and then clinical levels. Yet, the *in vitro* level, constituting the basis, must be established on firm ground. Detrimental intrinsic factors could be inconsistent sample withdrawals, poor documentation, incomplete *in vitro* testing of the donor material and many more.

In order to facilitate reproducibility and standardization of *in vitro* studies, and thus ultimately foster clinical translation, consensus on how PnD should be described, defined, and characterized in research studies is inevitable. We assessed the current situation in published literature using the PnD e-questionnaire.

The PnD e-questionnaire was completed by 35.5% of the SPRINT COST Action members. All entered data met the legal requirement of having a signed informed consent which clearly states that the donation is intended for research. Almost all (98.3%) respondents followed the “Declaration of Helsinki” that recommends that studies, involving human, human material and data, are to be judged by an ethics committee.

Regrettably, providing information on the status of the mother is far from possible for most researchers working with PnD. Due to frequently anonymized donations, hardly any information is released on the mother’s pre-existing medical history including pharmacological treatments during pregnancy. Information on lifestyle factors such as smoking, drug/alcohol abuse, or obesity is even less commonly obtained. However, this information may be key for the interpretation of the results, as it is known that these factors have substantial effects on cells and tissues.

For example, cigarette smoke extract was found to have profound effects on cellular processes in perinatal tissues and cells such as increase of reactive oxygen species (ROS) levels and DNA damage, thereby inducing cellular senescence (Menon et al., 2013). Cigarette smoke extract has also been shown to increase mitochondrial ROS (Polettini et al., 2014) or induction of inflammation by cytokine release via NFkappaB activation (Choltus et al., 2021). In general, cellular changes associated with senescence and/or with syndromes associated with early senescence, such as diabetes, also promote a pro-inflammatory state that weakens the fetal membranes and their functionality (Menon et al., 2019; Di Tomo et al., 2021). It is interesting to note that mesenchymal stromal cells (MSCs) from a different tissue source, such as human periodontal ligament-derived stem cells are also susceptible to cigarette smoke. Human periodontal ligament-derived stem cells, when derived from smokers, showed reduced proliferation and migration capabilities compared to corresponding cells from non-smokers (Ng et al., 2015). Taken together, these *in vitro* studies show that the regenerative potential of cells could clearly be affected by cigarette smoke extract.

Substances such as amphetamine (Jones et al., 2009), buprenorphine (Concheiro et al., 2009) or cocaine (Winecker et al., 1997) can be detected in the umbilical cord and are used as evidence for substance abuse. Conversely, if these substances accumulate in this specific tissue, it seems likely that they can also have an effect on the tissue.

Alcohol abuse is another issue of serious concern during pregnancy. Brien et al. found ethanol to be accumulated in the amniotic fluid (Brien et al., 1983). Cultured human umbilical vein endothelial cells (HUVECs) show significantly increased endothelial nitric oxide synthase activity and inhibited L-[3H]arginine uptake upon acute ethanol exposure (Acevedo et al., 2001). Furthermore, in a rat fetal alcohol exposure model, ethanol exposure resulted in significant alterations of the exosomal miRNA composition in amniotic fluid (Tavanasefat et al., 2020).

Not only external factors, but, obviously, also internal ailments can have effects on the fetus or the placental tissues. Obesity causes the rise of various inflammatory cytokines and matrix metalloproteinases (MMP) such as interleukin (IL)-1 β, IL-6, MMP-1, MMP-6 and MMP-13 (Melekoglu et al., 2018). Consequently, fetuses and fetal tissues of obese women are exposed to higher levels of pro-inflammatory cytokines and MMP levels compared to mothers with normal body weight (Melekoglu et al., 2018). It has also been shown that isolated human amniotic MSCs from obese women showed altered protein expression of metabolic pathways, stress response and cytoskeleton compared to those of women with normal body weight (Capobianco et al., 2016).

Gestational diabetes mellitus on the other hand also has profound effects on placental tissues. Maternal hyperglycemia, for example, lowers cell proliferation, angiogenesis, expression of stemness and differentiation markers, antioxidant enzymes, telomerase, and gene expression for mitochondrial function, and increases cell cycle inhibitors and p53 compared to those of healthy mothers (Kong et al., 2019; Pipino et al., 2022). According to another study, angiogenic properties of hAMSCs of mothers with gestational diabetes also change compared to healthy mothers (Klid et al., 2021).

When PnD are used only for research, tests for bacterial or viral infections of the donated tissue are not mandatory. This is clearly reflected in the results of the e-questionnaire, where providing information on the HIV/hepatitis B/hepatitis C/syphilis status, etc. often has no priority for PnD used in research settings. However, these factors can substantially alter the inflammatory status of the mother and again the properties of the tissues and consequently alter *in vitro* results. Therefore, knowledge on possible infections is crucial for the interpretation of results. In addition, the safety of the researcher, and possibly other personnel of the institution may be at risk, if potentially infectious tissue is handled without knowledge.

Another factor that could have a significant effect on the outcome of a study is the gender of the fetus. It is known that a number of cellular processes are different, depending on the sex of the donor (De Souza Santos et al., 2018). For example, MSC derived from Wharton’s Jelly showed significant gender-related differences regarding gene expression of stemness markers (Balzano et al., 2019). It is likely that this could have an effect on regenerative capacities of donor tissue and cells. In this regard, it is important to note that commonly used cell culture media often contain phenol red, which is known for its estrogen-like effect on cells (De Souza Santos et al., 2018). Yet, the results of the responses of the PnD e-questionnaire showed that only a quarter of the publications provided information on the gender of the fetus.

Taken together, these studies emphasize the importance of having comprehensive information on the donor. Although in some cases missing information may be due to the inexperience of the researcher, who may not consider the information relevant to downstream processes, in most cases it may be that day-to-day clinical practise or ethical concerns simply do not allow access to any, let alone meticulous, documentation of the mother’s health status. Justifiably, the welfare of the patient always comes first, and moreover, most hospitals painfully suffer from lack of medical staff. In addition, medical staff may not always be aware of the significance of research. On the other hand, the availability of donor tissues can also be an issue, and pressing deadlines of journals or grants often force researchers to accept any donor material that is available, regardless of how much information is provided on the donor.

Regarding information on tissue collection, all respondents explicitly referred to the specific tissue type from which the PnD were taken, however, information on the specific tissue regions was not always provided. This should be taken into consideration as there is some evidence, for example, for the amniotic membrane, that cells/tissues of different regions may have different regenerative potential (Centurione et al., 2018; Passaretta et al., 2020; Weidinger et al., 2020; Basile et al., 2023).

As many other fields in life science research, also PnD *in vitro* research strives to establish and maintain high rates of reproducibility of experimental procedures. Therefore, first, it is important to identify potential sources of variability in isolation/preparation/culture steps of PnD. This includes, but is not limited to, reagents/substrates/supplements and their concentrations, timing/duration of each isolation/preparation/culture step, temperature, washing steps, medium change, incubator environment (oxygen and carbon dioxide concentration), type of cell culture (adherent or in suspension), cell seeding density, and cell confluency. Second, it is crucial to precisely describe how all the steps of an experiment were performed. This would prevent what has been described in a *Nature* survey, according to which, up to 70% of experiments/studies are not reproducible, 60% of which not even by the original team (Baker, 2016). According to the results of the e-questionnaire, the standardization of the procedures and their documentation in PnD *in vitro* research is done fairly well in some areas, but there is a need for improvement in others.

Finally, to promote clinical translation, it is necessary to report information on the characterization of PnD. In particular, the focus lies on the functional *in vitro* characterization of PnD to demonstrate their potential efficacy and mode of action by monitoring, for example, cell proliferation, cell differentiation, cell death, immunomodulation, angiogenesis, migration, invasion or microbial growth. The data of the PnD e-questionnaire show that the respondents had different priorities in this regard. However, our SPRINT COST Action has recently published a quadrinomial series of consensus reviews to improve the quality of *in vitro* testing linked to i) inflammation, angiogenesis and wound healing (Flores et al., 2022), ii) oncological and antimicrobial applications (Silini et al., 2022), iii) immunomodulatory functions (Papait et al., 2022), and (iiii) PnD applications in brain, bone, skeletal muscle, heart, intestinal, liver, and lung pathologies (Pozzobon et al., 2022). These publications not only provide researchers with guidance on which tests are appropriate, but also discuss advantages and disadvantages of assessing functionality.

If human tissues and cells are intended for human applications, standards of quality and safety for the donation, procurement and testing are specified in Annex I/III of Directive 2006/17/EC (European Commission, 2006) (Supplementary Material). Besides minimizing the influence on the donor´s side, this guideline aims to ensure the safety of recipients of cells/tissues and guarantee standardization and reproducibility of experiments/products. If a tissue is used for research only, these guidelines do not apply. However, in order to promote clinical application, the donation, procurement, testing, processing, preservation, storage and distribution of human cells and tissues should be performed in accordance with Directive 2004/23/EC (European Parliament, 2004) and Directive 2006/17/EC (European Commission, 2006). In PnD research, there is often a lack of standardization throughout the process, from procurement to *in vitro* culturing and characterization, a problem that urgently needs to be addressed.

As a first step, the PnD e-questionnaire is meant to serve as the common basis for what information on materials and methods must be stated in publications. The PnD e-questionnaire is now available to the scientific community in order to guide researchers on the minimal criteria that should be clearly reported in their manuscripts. We invite PnD researchers to use the PnD e-questionnaire as a tool and guideline for performing and conducting experiments with PnD. The PnD e-questionnaire is available at the URL https://magic.elixir-hpc.si/PnD_invitro with access code “7DMYJJ9WN”. The PnD community will regularly update the PnD e-questionnaire as needed.

### 4.1 Limitations of the study

1. The absence of information on the mother’s medical history and lifestyle factors made it challenging to control potential confounding variables, which may bias and affect the accuracy of our findings.
2. Statistical analysis was performed using entries solely from the experts within the SPRINT COST Action, which does not encompass the entirety of the available literature on the subject.

Nevertheless, our aim is to transform the questionnaire into a widely accessible resource that will become more relevant as standardization in the field progresses.

## 5 Conclusion

Herein we aimed to critically assess how PnD are currently defined and characterized in published literature in order to define the minimal information that must be reported. This study reveals the broad variability in reporting quality and completeness in both PnD definition and product characterization. As standardization of methods is imperative for the reproducibility of results and comparability of studies, the PnD e-questionnaire should serve as the common basis for what information on materials and methods must be stated in PnD publications.

## Data Availability

The original contributions presented in the study are included in the article/Supplementary Material, further inquiries can be directed to the corresponding authors.
