# Supplementary material for: Recommendations from the COST action CA17116 (SPRINT) for the standardization of perinatal derivative preparation and in vitro testing
Source: Front Bioeng Biotechnol. 2023 Nov 14;11:1258753. doi: 10.3389/fbioe.2023.1258753 (PMC10682948; doi:10.3389/fbioe.2023.1258753)
Supplement: Supplementary file 1 [file DataSheet1.docx]

***Supplementary Material***

**Recommendations from the COST Action CA17116 (SPRINT) for the standardization of perinatal derivative preparation and *in vitro* testing**

**Aleksandar Janev**^†^**, Asmita Banerjee**^†^**, Adelheid Weidinger**^†^**, Jure Dimec, Brane Leskošek_,_ Antonietta Rosa Silini, Tina Cirman, Susanne Wolbank, Taja Železnik Ramuta, Urška Dragin Jerman, Assunta Pandolfi, Roberta Di Pietro, Michela Pozzobon, Bernd Giebel, Günther Eissner, Polonca Ferk, Ingrid Lang-Olip, Francesco Alviano, Olga Soritau, Ornella Parolini*, Mateja Erdani Kreft^*^**

^†^These authors have contributed equally to this work and share first authorship.

*** Correspondence:**Prof. Mateja Erdani Kreft, PhD

E-mail: [mateja.erdani@mf.uni-lj.si](mailto:mateja.erdani@mf.uni-lj.si)

Prof. Ornella Parolini, PhD

E-mail: ornella.parolini@unicatt.it

# Supplementary Data

## Legislative requirements regarding donor selection, tissue procurement and mandatory testing of donated PnD

When working with PnD, the first thing to do is – to identify and trace the potential donor. For EU member states the minimal selection criteria for donors of tissues and cells are specified in Annex I/III of Directive 2006/17/EC (European Commission, 2006) although individual member states can set additional criteria as local disease prevalence and risk assessments vary between countries and regions (European Commission, 2006; 2012; EDQM, 2022). When procuring PnD, the generic contraindications for tissue and cell donation are determined in Directive 2006/17/EC (European Commission, 2006) and explained in greater detail in the EDQM guide (EDQM, 2022). The explanation of relative contraindications, behavioral and personal risks that have to be taken into account when evaluating a potential donor are also explained there (EDQM, 2022). Beside general exclusion criteria that hold true for most of the donors, specific exclusion criteria for donors of placenta/fetal membranes are also described (the diseases of the female genital tract, significant local bacterial, viral, parasitic or mycotic infection of the genital tract, especially amniotic infection syndrome, (known) malformations of the unborn/newborn, premature rupturing of membranes; endometritis; meconium ileus) (EDQM, 2022).

After the donor is selected and before the donation takes place, an informed consent must be acquired. Since in the case of PnD the cells/tissues are procured during the planned surgical procedures, consent should be obtained before the planned delivery so interfering during the delivery is avoided. The donor of placenta/fetal membranes should also be informed that donation will only take place if the delivery is without any complications (EDQM, 2022). The consent must be specific and should include information about the processing, storage and intended use of the donated material with the scope and duration of the consent and (if the tissues/cells are to be stored) the information about the storage time and the fate of the cells/tissues once the period has expired. Consent cannot be assumed for uses of tissues or cells about which the donor has not been informed. The donor should also be informed about the tests to determine the suitability of the transplant. The consent to use donated cells/tissues for research application(s) must be signed separately from the consent for their use as a therapeutic product. Donors must also be made aware that they may withdraw consent at any time (EDQM, 2022).

Once the donor's consent/authorisation has been obtained, the procurement of human cells/tissues can take place. To protect the properties of cells/tissues that are required for their ultimate clinical use, as well as the safety of donors and recipients, the procurement organization or the tissue establishment undertaking the process must have a good quality system and be authorized by the appropriate and competent Health Authority (EDQM, 2022). This includes properly trained and qualified personnel, proper materials and equipment, appropriate packaging, labeling and transportation of procured tissue/cells to the processing facility. Where available, CE - marked devices and sterile single-use materials must be used for the procurement (EDQM, 2022). In EU member states, critical reagents and materials must meet documented requirements and specifications, and - when applicable - the requirements of Regulation (EU) 2017/745 (European Parliament, 2017) and Directive 98/79/EC (European Parliament, 1998). For the procurement of PnD specific measures must also be taken into account (EDQM, 2022). PnD should be collected only from living donors after a full-term pregnancy. As they could be contaminated by the normal vaginal flora during vaginal delivery, procurement in aseptic conditions after the elective cesarean section is preferred. If collection is performed during vaginal delivery, different sterilization procedures should be applied to the processed tissues/cells (EDQM, 2022).

All the donated PnDs must be tested as determined by national or other applicable legislation. For the EU, mandatory lab tests are specified in Annex II of Directive 2006/17/EC (European Commission, 2006) and amended by Directive 2012/39/EU (European Commission, 2012). Blood samples from the living donors must be collected and tested at the time of donation or - if not possible - within seven days post donation. Where tissues/cells of allogeneic living donors can be stored for long periods, repeat sampling and testing is required after 180 days (European Commission, 2006; 2012; EDQM, 2022). As NAT assays for HIV, HBV and HCV shorten the window period significantly, re-testing after 6 months is not required if NAT was performed (EDQM, 2022).

Blood samples from the living donors must be collected and tested at the time of donation or - if not possible - within seven days post donation. Where tissues/cells of allogeneic living donors can be stored for long periods, repeat sampling and testing is required after 180 days (European Commission, 2006; 2012; EDQM, 2022). As NAT assays for HIV, HBV and HCV shorten the window period significantly, re-testing after 6 months is not required if NAT was performed (EDQM, 2022).

In the case of neonatal donors (i.e., age ≤ 1 month), the biological tests may be carried out on the donor’s mother to avoid medically unnecessary procedures upon the infant. However, IgG antibodies in the newborn blood sample are likely to be maternal so caution when interpreting the results is needed (European Commission, 2006; 2012; EDQM, 2022).

## References

European Commission (2006). Commission Directive 2006/17/EC of 8 February 2006 implementing Directive 2004/23/EC of the European Parliament and of the Council as regards certain technical requirements for the donation, procurement and testing of human tissues and cells (Text with EEA relevance). *Official Journal of the European Union* 38**,** 40-52.

European Commission (2012). Commission Directive 2012/39/EU of 26 November 2012 amending Directive 2006/17/EC as regards certain technical requirements for the testing of human tissues and cells *Official Journal of the European Union* 327**,** 24-25.

European Parliament, C.O.T.E.U. (1998). Directive 98/79/EC of the European Parliament and of the Council of 27 October 1998 on in vitro diagnostic medical devices. *Official Journal of the European Union* 331**,** 1-37.

European Parliament, C.O.T.E.U. (2017). Regulation (EU) 2017/745 of the European Parliament and of the Council of 5 April 2017 on medical devices, amending Directive 2001/83/EC, Regulation (EC) No 178/2002 and Regulation (EC) No 1223/2009 and repealing Council Directives 90/385/EEC and 93/42/EEC (Text with EEA relevance.). *Official Journal of the European Union* 117**,** 1-175.

EDQM (2022). *Guide to the quality and safety of tissues and cells for human application.* Strasbourg, France: European Directorate for the Quality of Medicines & HealthCare (EDQM), Council of Europe.

European Commission (2006). Commission Directive 2006/17/EC of 8 February 2006 implementing Directive 2004/23/EC of the European Parliament and of the Council as regards certain technical requirements for the donation, procurement and testing of human tissues and cells (Text with EEA relevance). *Official Journal of the European Union* 38**,** 40-52.

European Commission (2012). Commission Directive 2012/39/EU of 26 November 2012 amending Directive 2006/17/EC as regards certain technical requirements for the testing of human tissues and cells *Official Journal of the European Union* 327**,** 24-25.

European Parliament, C.o.t.E.U. (1998). Directive 98/79/EC of the European Parliament and of the Council of 27 October 1998 on in vitro diagnostic medical devices. *Official Journal of the European Union* 331**,** 1-37.

European Parliament, C.o.t.E.U. (2017). Regulation (EU) 2017/745 of the European Parliament and of the Council of 5 April 2017 on medical devices, amending Directive 2001/83/EC, Regulation (EC) No 178/2002 and Regulation (EC) No 1223/2009 and repealing Council Directives 90/385/EEC and 93/42/EEC (Text with EEA relevance.). *Official Journal of the European Union* 117**,** 1-175.

# Supplementary Tables

**Supplementary Table 1.** Mandatory and other tests of donated cells/tissues.

| **Viral marker** | **Tests** |
| --- | --- |
| HIV 1 and 2 | Anti-HIV-1,2 (European Commission, 2006; 2012; EDQM, 2022)  HIV-1 p24 antigen strongly recommended (EDQM, 2022) |
| Hepatitis B | HBsAg (European Commission, 2006; 2012; EDQM, 2022)  Anti HBc (European Commission, 2006; 2012; EDQM, 2022) |
| Hepatitis C | Anti-HCV-Ab (European Commission, 2006; 2012; EDQM, 2022) |
| syphilis | A validated testing algorithm must be applied for the serological diagnosis (European Commission, 2006; 2012; EDQM, 2022) |
| additional testing | Depending on the donor’s history and the characteristics of the tissue or cells donated **(**for example ABO, RhD, HLA, malaria, antibodies to CMV, *Toxoplasma gondii*, EBV, *Trypanosoma cruzi*) (EDQM, 2022)  Determination of bacteraemia (EDQM, 2022)  Depending on other factors (individual travel history, specific current or past clinical abnormalities of the donor, epidemiological situation) - on a case-by-case basis: malaria, trypanosomiasis, viral infections with West Nile virus, Zika virus, HTLV-I antibody (EDQM, 2022) |
